# Complete NMR chemical shift assignments of odorant binding protein 22 from the yellow fever mosquito, *Aedes aegypti*, bound to arachidonic acid

**DOI:** 10.1007/s12104-019-09875-0

**Published:** 2019-01-25

**Authors:** David N. M. Jones, Jing Wang, Emma J. Murphy

**Affiliations:** 1 Department of Pharmacology, University of Colorado School of Medicine, 12801 East 17th Ave, Aurora, CO 80045 USA; 2 Program in Structural Biology and Biochemistry, University of Colorado School of Medicine, 12801 East 17th Ave, Aurora, CO 80045 USA

**Keywords:** *Aedes aegypti*, Odorant binding protein 22, Arachidonic acid, NMR resonance assignments

## Abstract

*Aedes aegypti* mosquitoes are the vector for transmission of Dengue, Zika and chikungunya viruses. These mosquitos feed exclusively on human hosts for a blood meal. Previous studies have established that Dengue virus infection of the mosquito results in increased expression of the odorant binding proteins 22 and 10 within the mosquito salivary gland and silencing of these genes dramatically reduces blood-feeding behaviors. Odorant binding proteins are implicated in modulating the chemosensory perception of external stimuli that regulate behaviors such as host location, feeding and reproduction. However, the role that AeOBP22 plays in the salivary gland is unclear. Here, as a first step to a more complete understanding of the function of AeOBP22, we present the complete backbone and side chain chemical shift assignments of the protein in the complex it forms with arachidonic acid. These assignments reveal that the protein consists of seven α-helices, and that the arachidonic acid is bound tightly to the protein. Comparison with the chemical shift assignments of the apo-form of the protein reveals that binding of the fatty acid is accompanied by a large conformational change in the C-terminal helix, which appears disordered in the absence of lipid. This NMR data provides the basis for determining the structure of AeOBP22 and understanding the nature of the conformational changes that occur upon ligand binding. This information will provide a path to discover novel compounds that can interfere with AeOBP22 function and impact blood feeding by this mosquito.

## Biological context

*Aedes aegypti* mosquitoes are responsible for the transmission of multiple diseases that including Dengue, chikungunya, Zika and yellow fever viruses. Whilst these diseases have been considered largely tropical in nature, the impact of climate change has dramatically increased the potential ranges for these mosquitoes into densely populated areas within the US and elsewhere. Disease transmission occurs when a female mosquito takes a blood meal from a human host. *Aedes aegypti*, in common with the malaria mosquito *Anopheles gambiae*, has evolved to feed exclusively on humans and this host selection is regulated by the perception of specific chemical cues that emanate from the human hosts (Braks et al. 1999). The presence of different chemical signatures directly impacts the attractiveness of an individual to the mosquito (Verhulst et al. 2013). It has been shown that mosquitoes infected with the malaria parasite *Plasmodium falciparum* exhibit increased attraction to human scent (Smallegange et al. 2013). Whilst humans infected with the parasite, are more attractive to mosquitoes (Lacroix et al. 2005).

Recently, it was shown that Dengue virus infection of *Ae. aegypti* could also potentially impact control of mosquito feeding behaviors (Sim et al. 2012). Viral infection was found to increase the expression of a set of chemosensory genes within the mosquito salivary gland, which included the odorant binding proteins (OBPs) 22 and 10 (AeOBP22 and AeOBP10) (Sim et al. 2012). Subsequently, it was demonstrated that knock down of these genes using siRNA approaches led to a significant reduction in the blood feeding behavior of these mosquitoes (Sim et al. 2012).

Odorant binding proteins are a highly abundant and diverse group of proteins (Hekmat-Scafe et al. 2002) that are generally found in the chemosensory tissues of insects (Shanbhag et al. 2001) where they can bind to a range of chemically related ligands(Campanacci et al. 2001; Honson et al. 2003; Pelosi et al. 2006; Plettner et al. 2000; Swarup et al. 2011). There is evidence for multiple roles of OBPs, including transporting ligands to the vicinity of odorant receptors (Damberger et al. 2000; Horst et al. 2001; Sandler et al. 2000; Wojtasek and Leal 1999; Ziegelberger 1995), activating odorant receptor complexes (Berg and Ziegelberger 1991; Laughlin et al. 2008; Pophof 2002, 2004; Xu et al. 2005), modulating sensitivity of odorant receptors (Larter et al. 2016) and terminating signal transmission (Vogt and Riddiford 1981; Ziegelberger 1995). AeOBP22 is expressed in multiple tissues, including the antenna, the salivary gland, the male reproductive tissues, and is transferred to females during mating (Li et al. 2008; Sim et al. 2012), suggesting that this OBP may regulate multiple behaviors that range from host seeking and feeding to reproduction. Therefore AeOBP22 is a novel target to potentially disrupt transmission o*f Ae. aegypti* borne infections and also control of mosquito populations.

Preliminary screens of potential ligands have revealed compounds that can bind to AeOBP22 in the high nanomolar to micromolar range (Li et al. 2008; Yang et al. 2011). However, there is no data that links these binding events to any changes in mosquito behavior. In order to better define the specific compounds that AeOBP22 can bind, and how this binding may regulate blood feeding, we have initiated structural and biochemical studies of the protein. In the process we discovered that AeOBP22 binds very tightly to lipids and fatty acids. Here, we present the backbone and side chain chemical shift assignments of AeOBP22 in the complex that it forms with arachidonic acid (AA). These results represent the first reported NMR chemical shift assignments of arachidonic acid in a complex with a protein, and only the second ever NMR solution based studies of a protein-arachidonic acid complex (Coudevylle et al. 2011). We show that AeOBPP22 binds arachidonic acid in a tight, stable complex, and we identify the region of the protein that undergoes a significant conformational change upon binding ligand. We propose that this conformational change regulates ligand binding and release, and a complete structural analysis will allow for the discovery of novel ligands that can disrupt ligand binding.

## Materials and methods

### Protein expression and purification

The majority of odorant binding proteins in insects are secreted proteins, and analysis of the *Ae. aegypti* OBP22 gene (Vector base AAEL005772) using the Signal-IP v 4.1 (Petersen et al. 2011) predicts a cleavage site for the signal peptide between residues 16 and 17 of the full length protein. We used *Ae. aegypti* cDNA (gift from Dr. Richard Vogt) to PCR amplify and subclone the gene fragment corresponding to the mature form of the protein ( residues 17–138) into the *NdeI* and *BamH1* restrictions site of the pET13a vector (Studier et al. 1990). This construct lacks the native signal peptide sequence but introduces an N-terminal initiator methionine; otherwise the vector contains no expression tags. We have numbered our assignments with the methionine considered as residue 1. The protein was expressed in *Escherichia coli* BL21(DE3) in cell cultures grown in minimal media supplemented with 2 g/L ^13^C D-glucose (> 99 atom %) and 1 g/L ^15^N NH_4_Cl (> 98 atom %) (Sigma Aldrich). Cells were grown at 37 °C to an OD_600_ of 0.5–0.6, and protein expression induced by addition of 1 mM isopropyl-1-thio-d-galactopyranoside (IPTG) and grown with shaking overnight at 25 ºC. The protein expressed in this way was purified from inclusion bodies essentially as previously described (Kruse et al. 2003; Murphy et al. 2013). The published protocol was modified in that the crude inclusion body pellet was washed three times with wash buffer 1 (20 mM Tris, pH 7.5, 0.5% Triton X-100, 1 mM EDTA and 1M Urea) followed by three times with wash buffer 2 (20 mM Tris, pH 7.5, 1 mM EDTA). The pellets were sonicated for 2 × 30 s during each wash step. The washed pellet was solubilized in 5 M guanidine hydrochloride, 5 mM dithiothreitol and 0.5 mM PMSF and the denatured protein was refolded using a cysteine/cystine redox reaction in the presence of 1% butanol, as previously described (Kruse et al. 2003; Murphy et al. 2013). The resulting protein solution was extensively dialyzed against sodium phosphate buffer (20 mM, pH 6.5), concentrated, and purified using size exclusion chromatography (Superdex S75) on an AKTA purifier system (Amersham Pharmicia Biotech). Purified protein, which eluted as a monomer, was checked for correct folding by circular dichroism (CD) spectroscopy (15 µM and at 25 °C) on a Jasco-815 spectropolarimeter in the CU School of Medicine biophysics core.

Samples of the complex between AeOBP22 and arachidonic acid (NuCheck Prep, Elysian MN) were made by addition of the arachidonic acid (in ethanol) to a final concentration of 200 µM to a sample of the protein at a concentration of 100 µM, and this was incubated overnight at 25 °C, concentrated and the buffer exchanged 3x to remove excess ethanol.

### NMR experiments

NMR experiments were recorded using ^15^N and ^13^C labeled protein (400–650 µM) in 20 mM sodium phosphate at pH 6.5, 10% D_2_O and 2,2,-dimethyl-2-silapentane-5-sulfonic acid (DSS) (80 μM) as the internal chemical shift reference. NMR experiments were performed at 25 °C on a Varian INOVA 600 MHz or a Varian/Agilent DD2 900 MHz spectrometer both equipped with cryoprobes. Assignments of the protein main-chain atoms were made using sensitivity enhanced versions of 2D ^1^H/^15^N-HSQC and 2D ^1^H/^13^C-HSQC (Kay et al. 1992), 3D HNCO (Grzesiek and Bax 1992b; Muhandiram and Kay 1994), 3D (HACA)CO(CA)NH (Lohr and Ruterjans 1995), 3D HNCACB and CBCA(CO)NH (Grzesiek and Bax 1992a), 3D CC(CO)NH (Grzesiek et al. 1993). While assignments of side chain ^13^C and ^1^H resonances were made using 3D HBHA(CBCACO)NH (Grzesiek and Bax 1993), 3D H(CCCO)NH (Grzesiek et al. 1993) and a simultaneous 3D ^15^N/^13^C- NOESY-HSQC (Vögeli et al. 2013). This latter experiment was modified from the published version to use broadband adiabatic inversion pulses on ^13^C during the INEPT periods. Aromatic protons were assigned from 2D (HB)CB(CGCD)HD and (HB)CB(CGCDCE)HE experiments (Yamazaki et al. 1993).

All 3D experiments were collected using non uniform sampling methods (Barna et al. 1987) using the Poisson-gap sampling schemes implemented by Hyberts et al. (Hyberts et al. 2010) and with a sampling density of 25–40%. Sampling densities were generally selected based on the expected dynamic range, with lower sampling densities being used in experiments with higher signal to noise and more uniform peak intensities, e.g. HNCO and CBCA(CO)NH where reconstruction artifacts were less likely to impact data interpretation, whilst NOESY spectra were acquired with the highest sampling densities. Data were processed using the istHMS package v2111 (Hyberts et al. 2014, 2012) in combination with NMRpipe (Delaglio et al. 1995) and resonance assignments were determined using Ccpnmr Analysis v 2.4.2 (Vranken et al. 2005).

The assignments of the arachidonic acid in the complex were made using 2D ^15^N/^13^C-filtered NOESY and TOCSY experiments (Ikura and Bax 1992; Zwahlen et al. 1997) (Fig. 2) recorded using a sample of the complex prepared in the same way but using 99% D_2_O containing sodium phosphate (20 mM, pH 6.1).

### Extent of the assignments for the OBP22-archidonic acid complex

**Fig. 1:**
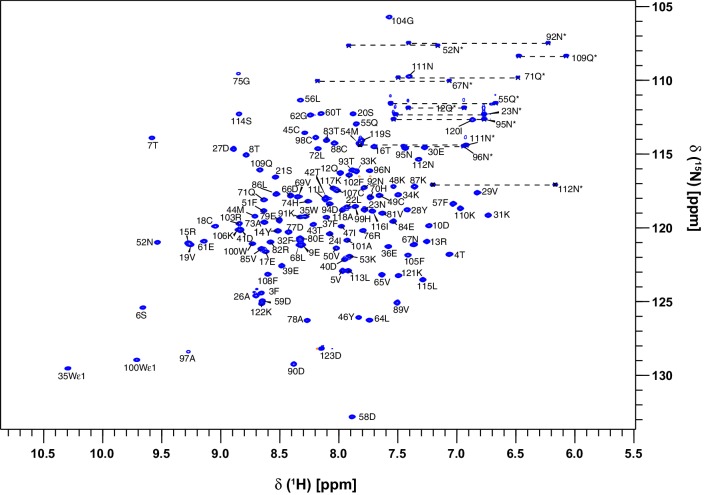
Backbone amide resonance assignment of AeOBP22 bound to arachidonic acid. The 2D [^1^H-^15^N] –HSQC spectrum of uniformly ^13^C^/15^N - labeled AeOBP22 recorded at 25 °C and at 900 MHz is shown and the peaks are labeled with the assignments. 100% of the non-proline residues could be assigned in this spectrum. Assignments of the side-chain NH2 groups from the Asn and Gln resides are indicated with an asterisk, and the pairs of protons are connected by dashed lines

The assignments of OBP22 in the complex with arachidonic acid were made manually within Ccpnmr’s Analysis program. (Vranken et al. 2005). In the ^1^H-^15^N HSQC experiment the assignments of the backbone amides are 100% complete, with the exception of the N-terminal methionine (Fig. 1). In addition, assignments for the Cα, Cβ, Hα and Hβ resonances are also 100% complete. The backbone CO assignments are 96% complete, 4 assignments could not be made unambiguously because of overlap. Assignments of the Hγ and Cγ resonances, excluding CγO and Cγ of aromatic residues, are also 100% complete. 95% of the Cδ resonances could be assigned (excludes CδO, and Cδ in Trp). The three missing assignments are the Cδ of Phe32 and Phe57, which are overlapped with other aromatic resonances, and Lys48, which is line broadened due to conformational exchange. The Hε and Cε resonances are 73% and 75% complete respectively. Here the notable absences are the Hε1 protons of the three histidines, which all exhibit significant line broadening, that prevents unambiguous assignment. Finally the H_Z_ and H_H_ resonances are 83 and 100% assigned respectively. Analysis of the chemical shift assignments, confirms that AeOBP22 is predominantly α-helical, in common with other insect OBPs (Fig. 3).

### Assignment of arachidonic acid

Only a single set of peaks are observed for the arachidonic acid resonances in the complex with OBP22. Further, the chemical shifts of the vinylic protons have significantly greater dispersion compared those observed in solution for the free fatty acid. ^1^H-^15^N-HSQC spectra recorded of the protein recorded using sub-stoichiometric concentrations of the lipid show two distinct sets of peaks (not shown), and these overlap with the peaks in the spectra of the free and bound states of the protein. Spectra recorded with increasing lipid concentration show a shift in the population of the peaks from the bound state and a reduction in the peaks from the free state, indicating that binding of the lipid is in slow exchange on the NMR timescale. This is in agreement with preliminary fluorescent based binding assays that suggest that arachidonic acid binds with a *K*_D_ of ~ 230 nM. We interpret the lack of any signal from the free state of the lipid in the samples used for our NMR assignment and structure determination as a result of micelle formation as the critical micelle concentration for arachidonic acid is reported to be in the range 10–60 µΜ (Pompeia et al. 2003; Serth et al. 1991).

**Fig. 2:**
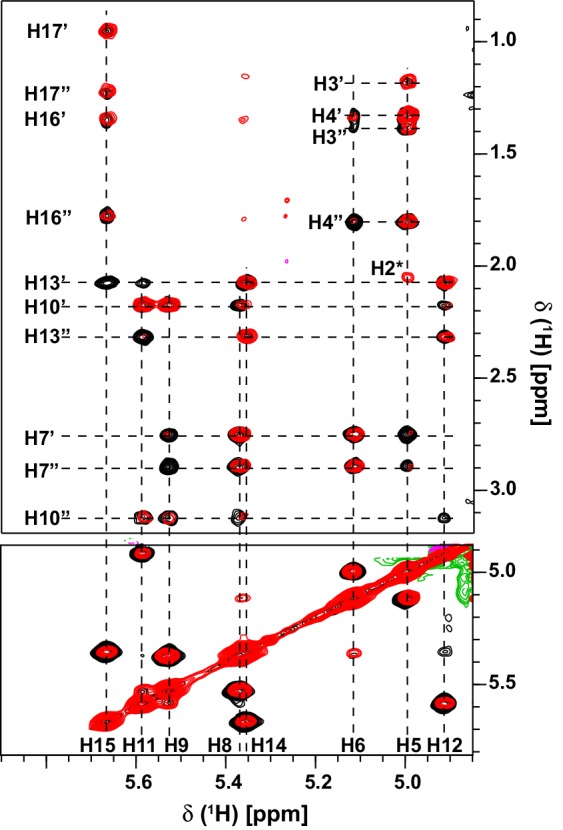
Resonance assignments of arachidonic acid bound to AeOBP22. Overlay of the vinylic proton region from the 2D-[^13^C/^15^N]-filtered TOCSY (black) and NOESY (red) spectra recorded using uniformly ^13^C^/15^N—labeled AeOBP22 and unlabeled fatty acid. The spectrum suppresses signals from the protein. The vinylic protons at each end of the double bond (labels at bottom) exhibit characteristic correlations to the remaining protons within the lipid chain (labeled vertically at left), enabling facile assignment for the lipid

The assignment of the arachidonic acid proton resonances was facilitated by the virtue that each vinylic proton was in a unique environment within the protein and these resonances show limited overlap with resonances from the protein. An overlay of the ^13^C-filtered NOESY and TOCSY experiments showing the vinylic proton resonances from the lipid in the region between 4.8 and 6.0 ppm is shown in Fig. 2. A single proton at 5.625 ppm exhibits correlations to multiple methylene protons in the range 0.8–1.7 ppm, establishing it as originating from H15. Two other vinylic protons, at ~ 5.0 and 5.12 ppm show correlations to methylene protons in the range 1.3–2.0 ppm including to the H2 position (immediately adjacent to the C1 carboxyl group), establishing these as the H5 and H6 protons respectively. Using these as the entry points it was possible to assign all the remaining protons in the lipid chain. That all the protons in the lipid chain have unique chemical shifts, reflects the unique interactions that must be formed with the protein. Insect OBPs generally bind to ligands within a central binding cavity, and intermolecular NOESY experiments confirm that the arachidonic acid is binding in the central pocket of OBP22.

### Backbone assignments of apo AeOBP22

**Fig. 3:**
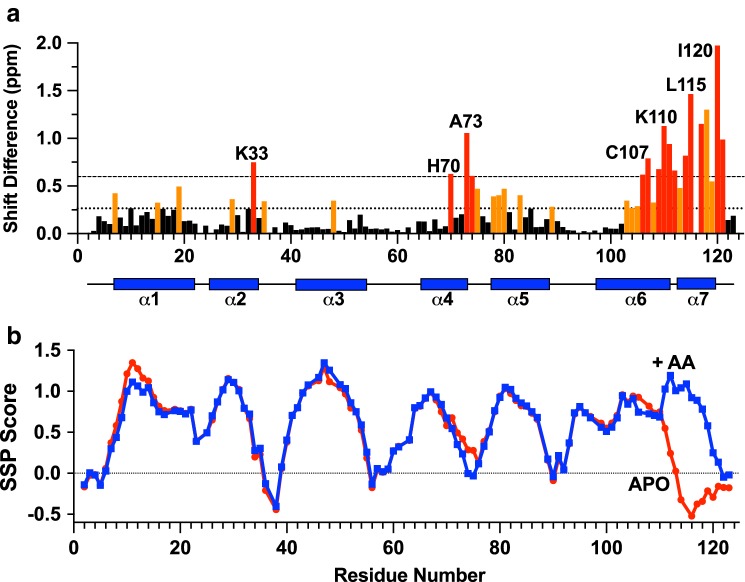
The C-terminal tail of AeOBP22 undergoes a conformational change on binding lipid. **a** Plot of the normalized chemical shift difference between AeOBP22 in the apo state and bound to arachidonic acid as a function of residue number. Normalized shift differences were calculated as Δδ = sqrt (ΔδH^2^ + 0.15*ΔδN^2^). Residues that show shift changes greater than the mean are colored orange and those that are greater than 1 s.d. above the mean are colored red. Horizontal dashed lines indicated the values for the mean shift change and the mean plus 1 s.d. **b** Plot of the secondary structure propensity calculated using SSP (Marsh et al. 2006) for the apo AeOBP22 (red) and arachidonic acid bound protein (blue). The location of the predicted alpha-helical regions in the bound state is shown in the panel above as blue bars. For the apo-protein there is a clear break in the predicted secondary structure at residue 111

In order to better understand the nature of any conformational changes that occur on ligand binding to AeOBP22 we also obtained backbone assignments for the H, N, Cα and Cβ resonances of the apo form of the protein. We have obtained assignments for 117 of the expected 119 amides for this sample. In the ^1^H-^15^N-HSQC spectrum of the apo protein we do not observe peaks for residues 2 and 116. There are large chemical shift differences between the apo and bound states of AeOBP22 in multiple locations in the protein, with the largest differences localized to the C-terminal residues 104–123 (Fig. 3a). A comparison of the calculated secondary structure propensities for the apo and bound states (Marsh et al. 2006) (Fig. 3b) reveals that the overall secondary structure of the protein is maintained throughout most of the protein between the apo and bound states. In contrast, the C-terminal residues are significantly less ordered in the apo state. This indicates that the binding of arachidonic acid may be regulated by a conformational change that occurs in the C-terminal region of the protein.

## Discussion

*Aedes aegypti* OBP22 is implicated in regulating the blood feeding behavior of this mosquito (Sim et al. 2012). However, the specific chemosensory stimulus(i) that is involved in this response is as yet unknown. In the process of trying to identify the natural ligands for AeOBP22, we have discovered that it binds tightly to fatty acids, including arachidonic acid. Here we have shown that the binding of arachidonic acid induces a significant conformational change in the C-terminal residues, suggesting that region of the protein may be responsible for regulating ligand binding. Further it is apparent that the arachidonic acid must adopt a highly defined conformation within the protein, as there is no evidence of chemical exchange for the lipid resonances. The question that arises is what functional role does AeOBP22 fulfill in binding arachidonic acid and/or other fatty acids. One hypothesis is that is recognizing specific lipids and delivering these to chemosensory receptors in the mosquito that are required to stimulate blood feeding. This would correlate with the observed reduction in feeding behaviors seen in dsRNA knock down of OBP22 gene expression (Sim et al. 2012). However, many fatty acids, including AA, are released from cell membranes by the activity of phospholipase A2. These can function both as intracellular messengers but also, as in the case of AA, as precursors of proinflammatory signals. Therefore, an alternative hypothesis is that AeOBP22 sequesters pro inflammatory signals as a mechanism to guard against premature termination of blood feeding.

Ongoing structural studies of AeOBP22 using the data presented here, will inform on the specific ligands that can interact with AeOBP22 and better inform on its biological function. Knowledge of the nature and magnitude of the conformational changes that take place when ligands bind will generate structural information that can used to discover novel compounds that have the potential to complete for ligand binding, disrupt OBP22 function, and impact the blood-feeding behavior of the mosquito.
