# Correction to: Complete NMR chemical shift assignments of odorant binding protein 22 from the yellow fever mosquito, *Aedes aegypti*, bound to arachidonic acid

**DOI:** 10.1007/s12104-019-09891-0

**Published:** 2019-04-02

**Authors:** David N. M. Jones, Jing Wang, Emma J. Murphy

**Affiliations:** 1 Department of Pharmacology, University of Colorado School of Medicine, 12801 East 17th Ave, Aurora, CO 80045 USA; 2 Program in Structural Biology and Biochemistry, University of Colorado School of Medicine, 12801 East 17th Ave, Aurora, CO 80045 USA

## Correction to: Biomolecular NMR Assignments (2019) 13:187–193 10.1007/s12104-019-09875-0

The article listed above was initially published with incorrect copyright information.

Upon publication of this Correction, the copyright of the article is changed to “The Author(s)”.

The original article has been corrected.

